# Activation of the sympathetic-adrenal-medullary system increases DNA damage during the transition to captivity

**DOI:** 10.1093/iob/obaf019

**Published:** 2025-05-09

**Authors:** D A V Kilgour, L M Romero

**Affiliations:** Department of Biology, Tufts University, Medford, MA 02155, USA; Department of Biology, Tufts University, Medford, MA 02155, USA

## Abstract

Prior work has demonstrated that both acute and chronic stress can increase the number of double-stranded breaks detected in DNA and that the hypothalamic-pituitary-adrenal axis is the primary driver of increases in DNA damage during acute stress. However, the role of the sympathetic-adrenal-medullary (SAM) system in causing the increase in DNA damage observed during chronically stressful situations such as the transition to captivity is less well understood. We tested the hypothesis that chronic SAM activation via catecholamine release increases DNA damage by administering a beta-blocker to wild house sparrows (*Passer domesticus*) at capture and throughout the day during the first few days of captivity. We quantified double-stranded DNA breaks throughout the 2-week transition to captivity. We found that immediately following the treatment period, both control and beta-blocker-treated birds had similar levels of DNA damage, but after 2 weeks in captivity, treated birds had lower levels of damage. These data suggest that SAM system activation plays a role in creating the previously observed patterns of DNA damage during chronic stress and that suppressing SAM effects may lead to faster recovery and less damage overall, thereby easing the transition to captivity for wild animals.

## Introduction

In daily life, wild animals may face a number of unexpected or unpredictable stimuli, such as predator attacks or sudden, extreme weather events, that are called stressors. Immediately after encountering a stressor, the physiological stress response is activated to help the animal respond to and recover from the stressor (Sapolsky et al. 2000; Romero and Wingfield 2016). The sympathetic-adrenal-medullary (SAM) system is activated first, leading to the release of catecholamines (e.g., epinephrine) within seconds of encountering the stressor to enable the typical fight-or-flight response (Romero and Wingfield 2016). Simultaneously, the hypothalamic-pituitary-adrenal (HPA) axis is activated, which results in the release of glucocorticoids from the adrenal glands within minutes of encountering the stressor (Sapolsky et al. 2000; Romero and Wingfield 2016). This response to acute stressors is crucial for survival and recovery; however, when stressors are repeated or ongoing, it can result in chronic elevations of the physiological mediators involved, leading to dysregulation of the stress-response systems that begin to negatively impact the animal's health (Sapolsky et al. 2000; McEwen and Wingfield 2003; Romero et al. 2009).

One well-known cause of chronic stress is holding wild animals in captivity (Terio et al. 2004; Dickens et al. 2009; Lattin et al. 2012; Love et al. 2014; Fischer et al. 2018; Fischer and Romero 2019; Marino et al. 2020). There are several reasons that wildlife may be brought into captivity for varying lengths of time, including for research, rehabilitation, captive breeding, or translocation. This transition introduces a myriad of new stressors that the animals cannot escape, such as artificial light and sounds, strange odors, restricted movement, forced proximity to humans, changes to diet, and non-natural social structures and interactions (Morgan and Tromborg 2007). These stressors often result in increased activation of the SAM system and HPA axis that leads to a variety of issues, including weight loss, changes to heart rate, altered glucocorticoid release and negative feedback, changes to the reproductive system, and altered immune function (Millar and Threadgill 1987; Ruiz et al. 2002; Terio et al. 2004; Dickens et al. 2009; Dickens and Romero 2009; Adams et al. 2010; Kuhlman and Martin 2010; Lattin et al. 2012; Love et al. 2014; Dickens and Bentley 2014; Lèche et al. 2016; Fischer et al. 2018; Taff et al. 2018; Fischer and Romero 2019; Marino et al. 2020). On a molecular level, chronic stress can also lead to an increase in DNA damage in the form of double-stranded DNA (dsDNA) breaks, which accumulate throughout the transition to captivity (Gormally, Fuller, et al. 2019). The accumulation of DNA damage can have both immediate and lasting consequences by impeding the body's ability to transcribe vital genes and is linked to pathologies such as tumor growth and cognitive decline (Gidron et al. 2006; Hoeijmakers 2009; Bonisoli-Alquati et al. 2010; Flint and Bovbjerg 2012; Flint et al. 2013).

While both acute (Gidron et al. 2006; Malandrakis et al. 2016; Gormally et al. 2020; Beattie et al. 2024) and chronic (Flint et al. 2013; Gormally, Fuller, et al. 2019; Naz et al. 2020; Beattie et al. 2024) stress have been linked to increased DNA damage, the respective roles of the SAM system and the HPA axis in causing this damage are still under investigation. *In vitro* exposure to corticosterone (the primary glucocorticoid in birds), epinephrine, and norepinephrine (catecholamines) can all increase DNA damage and interfere with damage repair mechanisms (Flint et al. 2007, 2013; Hara et al. 2011; Flaherty et al. 2017). *In vivo*, HPA axis activation appears to be the primary driver of DNA damage in wild-caught captive birds during the acute stress response, as blocking the receptors for glucocorticoids decreased damage, but blocking catecholamine receptors did not (Beattie et al. 2024). In comparison, blocking or knocking out the pathway stimulated by catecholamines during chronic restraint stress in captive-bred mice prevented increased DNA damage (Hara et al. 2013). However, uncertainty remains about how SAM system activation and catecholamine release increase DNA damage in wild animals and what drives the accumulation of DNA damage during chronically stressful conditions.

We hypothesized that activation of the SAM system plays a role in the amount of DNA damage detected during the transition to captivity. To test this hypothesis, we administered a beta-blocker—propranolol—during the transition to captivity to suppress the effects of the catecholamines by blocking the beta-adrenergic receptors for epinephrine (adrenaline) and norepinephrine (noradrenaline). While we know that *in vitro*, adding a beta-blocker with a catecholamine will prevent the increase in DNA damage observed with a catecholamine alone (Flint et al. 2007), we are less sure about its effect on DNA damage *in vivo* under chronically stressful conditions. We predicted that this treatment would decrease the amount of DNA damage detected during the transition to captivity. Specifically, we predicted that treated birds would (1) accumulate less DNA damage while receiving the beta-blocker during the first few days of captivity, (2) have lower levels of DNA damage at the end of the first 2 weeks in captivity, and (3) show a less prominent increase in DNA damage during the first 2 weeks in captivity. Overall, if the SAM system induces DNA damage during chronically stressful conditions like the transition to captivity, blocking its effects should result in a “flattening of the curve” of DNA damage over time that we have observed in previous research (Gormally, Fuller, et al. 2019).

## Methods

### Experimental design

We captured 16 house sparrows using mist nets and Potter traps between November 2022 and June 2024. The capture period was prolonged due to extenuating circumstances surrounding capture locations and researcher availability. Individuals were assigned to either the control group (*n* = 4 males, 4 females) or the treatment group (*n* = 4 males, 4 females) at the time of capture. Individuals caught during the breeding and non-breeding seasons were split nearly evenly between the groups (control: *n* = 2 non-breeding, 6 breeding; treatment: *n* = 3 non-breeding, 5 breeding) as season can affect DNA damage levels (Beattie et al. 2022). Within 5 min of capture, a small blood sample (<35 µL) was collected via puncture of the alar vein and collected in a heparinized capillary tube. Control birds were injected intramuscularly with sterile saline solution, while treatment group birds received a 3 mg/kg propranolol injection. This dosage is based on prior validations completed in our lab and has been used to block the effects of catecholamines and SAM system activation (Cyr et al. 2009; Fischer and Romero 2016; Beattie et al. 2024). Propranolol (Sigma–Aldrich Cat. No. P0884) was reconstituted in sterile saline at a concentration of 7.5 mg/mL. Initial bird weight was used to calculate the injection volume for both treatment and control groups, such that a 25 g bird received a 10 µL injection.

**Fig. 1.**
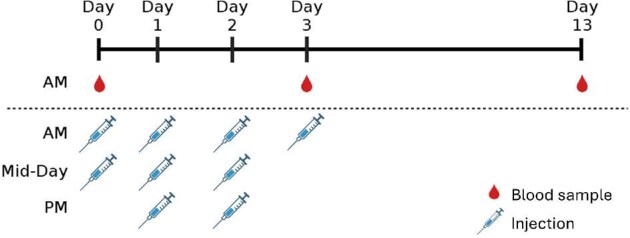
Summary of experimental design. Individuals received injections at capture and during the first 3 days of captivity. Blood samples were collected prior to injection at capture and at the end of the treatment period. A final blood sample was collected after 2 weeks of captivity.

The birds were then transferred to captivity, where they received a second injection before being placed into cages. Most individuals were doubly housed, while 2 males and 2 females had to be singly housed. However, cages were kept in close proximity so that singly housed individuals were always in visual and auditory contact with conspecifics. Birds were held on a 12L:12D light cycle and provided with *ad libitum* seed, grit, and water. On the first and second full days in captivity, birds received 3 more injections, once in the morning, at mid-day, and in the afternoon. On the third day, we collected another blood sample and administered a final injection (Fig. 1). Prior work demonstrated that one injection of propranolol a day on days zero, one, and two blocked the typical increase in corticosterone and decreased heart rate immediately following injection but did not have an effect on heart rate during the first week in captivity (Fischer and Romero 2016). As a result, we chose to increase the frequency of injections and target them to the first days of captivity when the increase in DNA damage is typically the most rapid (Gormally, Fuller, et al. 2019). We collected our last blood sample on day 13, after 2 weeks in captivity. This time point is where we would expect to see a peak in DNA damage based on prior work (Gormally, Fuller, et al. 2019). All work was approved by the Tufts University Institutional Animal Care and Use Committee and was conducted in compliance with the Guidelines to the Use of Wild Birds in Research (Fair et al. 2023).

### Comet assay

We used single-cell gel electrophoresis, also known as the comet assay (Collins 2004; Afanasieva and Sivolob 2018), to measure dsDNA breaks in red blood cells following the methods of (Gormally, Fuller, et al. 2019). Birds have nucleated red blood cells, and the background DNA damage present in blood samples is representative of damage in other tissues of the body (Beattie et al. 2022). Briefly, 2 µL of whole blood was combined with 800 µL of chilled phosphate-buffered calcium- and magnesium-free saline (PBS). Samples were then serially diluted 1:4 in fresh PBS twice more. Thirty microliters of each sample was then combined with 300 µL of low-melt agarose (R&D Systems Cat. No. 425005001), vortexed, and then 30 µL was added to a pre-coated CometSlide (R&D Systems Cat. No. 425250001) in duplicate. The slides were allowed to solidify at 4°C, then submerged in chilled lysis solution (mixed with 10% dimethyl sulfoxide; R&D Systems Cat. No. 425005001) for 1 h. Slides were removed from the lysis solution and submerged in chilled electrophoresis buffer (300 mM sodium acetate, 10 mM Tris base, pH 10) at 4°C for 30 min, then electrophoresed at 21 V, 90 mA for 30 min at 6°C. Finally, slides were washed with chilled deionized water twice, then 70% ethanol, and dried at 37°C. All steps to this point were completed in low lighting to avoid causing additional damage from UV radiation, and slides were stored in the dark with desiccant prior to imaging.

Slides were stained with SYBR Gold (Invitrogen Cat. No. S11494) for 30 min, rinsed with cold deionized water, dried at 37°C, and then imaged using a fluorescent microscope with a green fluorescent protein filter at 10× objective. Images were analyzed using the OpenComet plugin for ImageJ (Gyori et al. 2014), which automatically detects comets based on the intensity of their pixels and their shape. The algorithm then separates the head of the comet based on either the brightest region or the intensity profile of the pixels in the comet; the remaining region, following the head, is classified as the tail of the comet. The plug-in then calculates and outputs a variety of metrics based on the pixels contained in the image and their intensity. Every image was checked for erroneous comet detection before the final analysis by OpenComet. We used the tail moment metric calculated by OpenComet to quantify DNA damage, which is calculated as the proportion of DNA in the tail of the comet multiplied by the length of the comet tail (Gyori et al. 2014).

### Statistical analysis

All statistical analysis was completed in R version 4.4.1 (R Core Team 2024) using RStudio version 2024.9.0.375 (Posit team 2024). We used the “anova_test” function (rstatix package; Kassambara 2023) to calculate a mixed design analysis of variance (ANOVA) to test for within- and between-group differences in mean tail moment on days three and thirteen. The within-group differences are comparisons of the repeated measures taken on days three and thirteen, while the between-group differences are comparisons between the treatment and control groups. This test provides information on the effects of the within- and between-group factors alone, as well as the interaction between them. The anova_test function includes a specification for subject ID and accounts for possible correlations in the repeated measures taken from the same individuals. Data were checked for normality (Shapiro–Wilk test; *W *= 0.98, *P *= 0.46), homogeneity of variances (Levene's test; day zero: *F*_1,14_ = 0.20, *P *= 0.66; day three: *F*_1,14_ = 0.09, *P *= 0.77; day thirteen: *F*_1,14_ = 0.02, *P *= 0.88), and homogeneity of covariances (Box's *M* test; *M *= 0.79, *P *= 0.37). We also checked for an effect of breeding status on the tail moment and found that it did not alter our results. We used *post-hoc* unpaired *t*-tests with a Bonferroni *p*-adjustment to check for pairwise differences between groups and paired *t*-tests with a Bonferroni *p*-adjustment to check for pairwise differences within groups. All means are shown ± standard error.

## Results

**Fig. 2.**
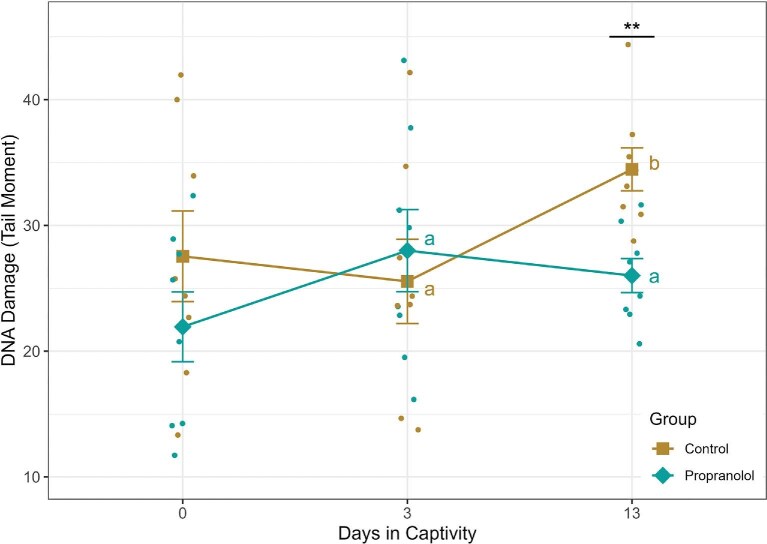
DNA damage in the propranolol-treated group did not increase from day three to day thirteen, while DNA damage was significantly higher on day thirteen in the control group. Letters indicate significant differences within groups. DNA damage was not significantly different between the treatment and control groups on day three, but the propranolol-treated group had fewer dsDNA breaks by day thirteen. Asterisks indicate significant differences between groups. Points are individual measurements, diamonds and squares represent group means, and error bars are the standard error of the mean.

The tail moment for the control group on day zero was 27.5 ± 3.61, on day three was 25.6 ± 3.35, and on day thirteen was 34.5 ± 1.71 (Fig. 2). The tail moment for the propranolol-treated group was 21.9 ± 2.78 on day zero, 27.4 ± 3.33 on day three, and 26.0 ± 1.36 on day thirteen (Fig. 2). While the main effect of the treatment group was not significant (mixed ANOVA, Treatment: *F*_1,14_ = 1.78, *P = *0.20), the main effect of day and the interaction of the treatment group and day approached the significance threshold of 0.05 (Mixed ANOVA, Day: *F*_2,28_ = 2.79, *P = *0.08; Treatment*Day *F*_2,28_ = 2.89, *P = *0.07).

When day zero was excluded from the analysis due to high individual variation reflecting prior conditions rather than experimental treatment, the main effects of the treatment group and day alone were not significant (mixed ANOVA, Treatment: *F*_1,14_ = 1.21, *P = *0.29; Day: *F*_1,14_ = 2.04, *P = *0.18), but there was a significant interaction between treatment and day (mixed ANOVA, *F*_1,14_ = 5.06, *P = *0.04). On day three, there was no significant difference between the propranolol-treated birds and the control birds (Fig. 2; *post-hoc* independent samples *t*-test, *t*_14_*= −*0.52, *P = *0.61), but by day thirteen, the control birds had significantly more dsDNA breaks (Fig. 2; *post-hoc* independent samples *t*-test, *t*_13.3_ = 3.87, *P = *0.002). One control individual on day thirteen appeared to be an outlier that may have biased the result. However, removing this point did not affect the difference between the groups (*post-hoc* independent samples *t*-test, *t*_12.8_ = 4.02, *P *= 0.001). When compared within groups between days three and thirteen, dsDNA breaks increased significantly in the control group (Fig. 2; paired *t*-test, *t*_7_ = −4.42, *P = *0.003), while they did not change in the propranolol-treated group (Fig. 2; paired *t*-test, *t*_7_ = 0.60, *P* = 0.57).

## Discussion

We tested the hypothesis that the activation of the SAM system leads to an increase and accumulation of DNA damage during chronically stressful conditions, such as the transition to captivity. We treated wild-caught house sparrows with a beta-blocker at capture and during the first 3 days of captivity. We predicted this would result in lower levels of DNA damage throughout the transition period. While we expected that individuals would accumulate less DNA damage during treatment with the beta-blocker, our results indicated no significant difference between the groups at the end of treatment on day three. Interestingly, the treated group did have significantly lower levels of DNA damage at the end of the first 2 weeks in captivity, supporting our second prediction. Finally, when comparing DNA damage within the groups over time, our results support the prediction that the treatment would result in a less prominent increase in damage over the first 2 weeks in captivity. DNA damage in individuals in the control group significantly increased from day three to day thirteen, while damage in the treatment group did not change. Together, these results support the hypothesis that activation of the SAM system can affect the amount of DNA damage detected during the transition to captivity.

Prior research found an increase in DNA damage within the first 3 days of captivity that peaked around day ten before plateauing (Gormally, Fuller, et al. 2019). Our control group showed a similar pattern with an increase in damage from day three to thirteen. However, we did not observe an increase from day zero to day three. It is also important to note that the trends and differences we observed were only statistically significant when the at-capture measurements were omitted from the analysis. When including these measurements, the interaction and within-group differences were nearly significant (*P < *0.08). The DNA damage measured in the at-capture samples reflected the conditions experienced by that individual before sample collection, which could influence DNA damage. For instance, individuals were likely exposed to stressors prior to capture, including acute and perhaps chronic stressors that could increase damage (Gidron et al. 2006; Flint et al. 2013; Malandrakis et al. 2016; Gormally, Fuller, et al. 2019; Gormally et al. 2020; Beattie et al. 2024). Alternatively, increased DNA damage during early captivity could be related to increased activity during the initial adjustment to captivity, as more active captive budgerigars (*Melopsittacus undulatus*) had more single-stranded DNA breaks (Larcombe et al. 2015). We are still uncertain about how long DNA damage may persist after exposure to an acute stressor, and changes in DNA damage following a recovery period after chronic stress have been inconsistent (Gormally, Estrada, et al. 2019; Beattie et al. 2024). However, after 2 weeks in captivity, these inherent individual differences should lessen as a result of entering a common housing environment.

Nonetheless, the high level of variability in the at-capture samples, likely reflecting previous conditions, and the lack of effect during treatment supports the idea that there is a lag in DNA repair and the replacement of damaged red blood cells (Gormally, Estrada, et al. 2019). The life span of avian red blood cells is typically 35–45 days (Beuchat and Chong 1998), and older, more damaged red blood cells are constantly being removed and replaced by younger, relatively undamaged cells (Arias and Arias 2017). However, highly damaged cells likely accumulate during the early stages of captivity (Gormally, Fuller, et al. 2019), so treatments that reduce this initial accumulation may be critical to ameliorating the adverse health outcomes, including tumor growth, resulting from the interruption of transcription and translation of key genes typically caused by this lag in repair and replacement and the associated accumulation of DNA damage.

Our results suggest that the SAM system plays a role in the accumulation of damage under chronically stressful conditions. This finding is supported by prior work *in vitro* that found both acute and chronic exposure to catecholamines increased DNA damage in rodent and human cells, but pre-treatment with a beta-blocker prevented this increase (Flint et al. 2007, 2013; Hara et al. 2011; Sun et al. 2015; Lamboy-Caraballo et al. 2020; Valente et al. 2021). Furthermore, exposure to catecholamines *in vitro* and *in vivo* in mice reduced DNA repair ability by reducing the levels and functionality of a key DNA repair protein, p53 (Hara et al. 2011). Chronic restraint stress in mice produced similar results through reduced levels of the same DNA repair protein, leading to an accumulation of DNA damage (Feng et al. 2012; Hara et al. 2013). These effects could be nullified by administering a beta-blocker or knocking out the gene activated by binding the β_2_-adrenergic receptors (Hara et al. 2013). Jenkins *et al.* (2014) present a thorough review and description of the theoretical pathway by which binding of the β_2_-adrenergic receptors may result in this damage through increased production of cyclic adenosine monophosphate and protein kinase A that, in turn, lead to increased oxidative phosphorylation and the production of reactive oxygen species (ROS). The concentration of ROS overwhelms the normal protective antioxidant capacity, resulting in DNA damage. Concurrently, activation of the protein MDM2 increases due to activation of the phosphoinositide-3-kinase/Akt pathway. This activation leads to a loss of function of p53 and reduced DNA damage repair ability (Jenkins et al. 2014). This pathway is supported by other work, which has found that adrenergic stimulation led to the accumulation of ROS *in vitro* (Sun et al. 2015) and that an injection of epinephrine increases oxidative stress in rats (Salyha 2023).

While our work focused on the effects of chronic stress on dsDNA breaks, chronic stress may also alter other metrics of damage, including ROS and antioxidant balance, markers of oxidative damage and repair, and telomere shortening. For example, exposing Eurasian blackbirds (*Turdus merula*) to repeated immune and disturbance stressors for a year resulted in shortened telomeres and increased oxidative damage (Hau et al. 2015). Similarly, exposure to chronic predation risk increased oxidative damage in damselfly (*Coenagrion puella*) larvae, likely as a result of increased metabolic activity (Janssens and Stoks 2014). Interestingly, adaptation to the local environment may affect the degree of damage that occurs. For example, Eurasian blackbirds from rural habitats demonstrated a marked increase in oxidative damage in response to repeated stressors over the course of a year, while urban blackbirds showed an overall decrease in oxidative damage (Costantini et al. 2014). However, as all of our study birds were collected from urbanized areas, it is unlikely that capture location would affect our results. Finally, DNA damage can lead to increased cell senescence, and prior work has found that chronic social stress in mice (Lyons et al. 2025) and chronic psychosocial stress in humans (Rentscher et al. 2019) leads to an increase in the expression of the cell senescence gene p16^INK4a^. However, there was no evidence of telomere shortening as a result of chronic stress in humans (Rentscher et al. 2019).

Previous research has established that the transition to captivity can result in several changes to the activity of the SAM system. For example, captive Rocky Mountain bighorn sheep (*Ovis canadensis canadensis*; Coburn et al. 2010) and captive harbor porpoises (*Phocoena phocoena*; Siebert et al. 2011) both show lower concentrations of epinephrine and norepinephrine in response to capture when compared to free-living individuals. Additionally, heart rate in European starlings (*Sturnus vulgaris*) is elevated upon introduction to captivity but decreases within a day (Dickens and Romero 2009). In house sparrows, heart rate is also elevated early during the transition to captivity and takes almost 3 weeks to plateau (Fischer et al. 2018; Fischer and Romero 2019). Additionally, there are changes to heart rate variability, often used as a metric of sympathetic and parasympathetic nervous system control of heart rate. Alterations to heart rate variability are good indicators of both acute and chronic stress in humans (Kim et al. 2018; Immanuel et al. 2023) and animals (von Borell et al. 2007; Wascher 2021). However, the direction of the relationship between heart rate variability and chronic stress in humans is often inconsistent, possibly due to the variety of metrics used to report stress and quantify variability (Reviewed in Immanuel et al. 2023). However, Schubert *et al.* (2009) found decreased heart rate variability during chronic stress, similar to what is observed in wild birds. In wild-caught birds, heart rate variability is typically lower early in the transition to captivity, indicating SAM activation, and increases over the next week or so (Dickens and Romero 2009; Fischer and Romero 2016; Fischer et al. 2018), indicating recovery. This suggests increased sympathetic nervous system activity and higher concentrations of epinephrine and norepinephrine early during the transition. Studies in sheep (Stubsjøen et al. 2015) and rats (Sgoifo et al. 1997; Park et al. 2017) have also found increased sympathetic activity during chronic stress. In contrast, while heart rate variability was altered during lameness in dairy cows, the alterations reflected decreased sympathetic activity. However, this decrease could be attributable to lower locomotor activity in lame animals (Kovács et al. 2015). Increased sympathetic activity and the resulting increase in catecholamine release during the first days to a week of captivity are consistent with trends in DNA damage found in our results and previous research, where DNA damage also increased during the first weeks in captivity (Gormally, Fuller, et al. 2019). However, by blocking the beta-adrenergic receptors for the catecholamines, we were able to limit the amount of damage that accumulated at the end of 2 weeks.

Glucocorticoids can also increase DNA damage *in vitro* (Flint et al. 2007), and the HPA axis, rather than the SAM system, appears to drive DNA damage during acute stress (Beattie et al. 2024). Furthermore, the 2 systems interact such that epinephrine and norepinephrine trigger the release of adrenocorticotropic hormone, stimulating the release of glucocorticoids from the pituitary, and glucocorticoids can increase secretion of and responsiveness to catecholamines, making it difficult to tease apart these mediators under stressful conditions (Axelrod and Reisine 1984; Sapolsky et al. 2000; Adameova et al. 2009). For example, administering propranolol to house sparrows on days zero, one, and two of captivity led to reduced heart rate for 15 min after administration and blocked the typical increase in glucocorticoids after a week in captivity (Fischer and Romero 2016), corresponding with the connection between the systems. However, a later study found that DNA damage and glucocorticoids were not directly correlated during the transition to captivity in house sparrows (Gormally, Fuller, et al. 2019). In contrast, chronic treatment with glucocorticoids alters antioxidant capacity and increases the amount of ROS, often leading to increased oxidative stress and damage (Lin et al. 2004; Costantini et al. 2008, 2011). However, during the transition to captivity, treatment with a beta-blocker did not prevent weight loss (Fischer and Romero 2016), suggesting that a reduction in glucocorticoids alone was not sufficient to alleviate the effects of this type of chronic stress. Furthermore, while the use of a beta-blocker may have affected heart rate for short periods of time, the treatment did not seem to result in any changes to SAM system activity during the first week in captivity (Fischer and Romero 2016). It is possible that treatment with propranolol in this earlier study may not have occurred frequently enough during the transition to elicit a response. In contrast, we administered 9 doses of propranolol over the first 4 days of captivity (Fig. 1), which may have suppressed the effects of the SAM system more effectively and resulted in the observed reduction in DNA damage. Alternatively, the effects may have been delayed, like those in our propranolol-treated group, and were more important for recovery and faster acclimation than immediate changes.

Our findings support the role of the SAM system in causing DNA damage during chronically stressful conditions such as the transition to captivity. Administering a beta-blocker and suppressing the effects of the SAM system appears to help “flatten the curve” by decreasing the amount of DNA damage that accumulates and lowering the peak level of damage that occurred during the first 2 weeks of captivity. The effects of the beta-blocker treatment may be delayed. Still, they could be beneficial by limiting the accumulation of damage, which may lead to faster acclimation and recovery. In addition, improving our understanding of the dynamics of DNA damage and repair and the replacement of damaged cells will improve our understanding of why damage may persist, how pathology occurs, and what treatments are most effective at ameliorating the negative consequences of chronic stress. Teasing apart the specific roles of catecholamines and glucocorticoids in inducing this damage would provide additional insight into the mechanisms driving these trends. Finally, our findings are specific to the transition to captivity as a chronic stressor. Thus, expanding this work and exploring the roles of the SAM system and HPA axis in driving DNA damage under different types of chronically stressful conditions will be necessary. In conclusion, accumulating evidence indicates that administering a beta-blocker during the first few days of introduction to captivity can make the transition less injurious.

## Supplementary Material

obaf019_Supplemental_Files

## Data Availability

Data and code underlying this work are available in the online supplementary material.
