# Supplementary material for: Activation of the sympathetic-adrenal-medullary system increases DNA damage during the transition to captivity
Source: Integr Org Biol. 2025 May 9;7(1):obaf019. doi: 10.1093/iob/obaf019 (PMC12151017; doi:10.1093/iob/obaf019)
Supplement: obaf019_Supplemental_Files [file obaf019_supplemental_files.zip › Read me - Metadata to accompany Table S1.docx]

Sample_ID - Identifier used to differentiate blood samples

Bird_ID - Identifier used to differentiate individuals

Capture_date - Date the individual was captured (MM/DD/YYYY)

Capture_time - Time of day at which the individual was captured (HH:MM)

Location - Location where the individual was captured

Sex - M = male, F = female

Group - Was the individual assigned to the treatment or control group

Date - Date the blood sample was collected (MM/DD/YYYY)

Time - Time of day the blood sample was collected (HH:MM)

Time_to_Bleed - Time since capture or disturbance to collect blood sample (M:SS)

Injection - Did the individual receive an injection at this time? Y=Yes, N=No.

Day - Day of captivity/the experiment

TailMoment - Metric used to quantify DNA damage in this analysis

Remaining columns contain additional metrics that could be used the quantify DNA damage. See Table 2 from Gyori et al. 2014 below for a description of how each is calculated.


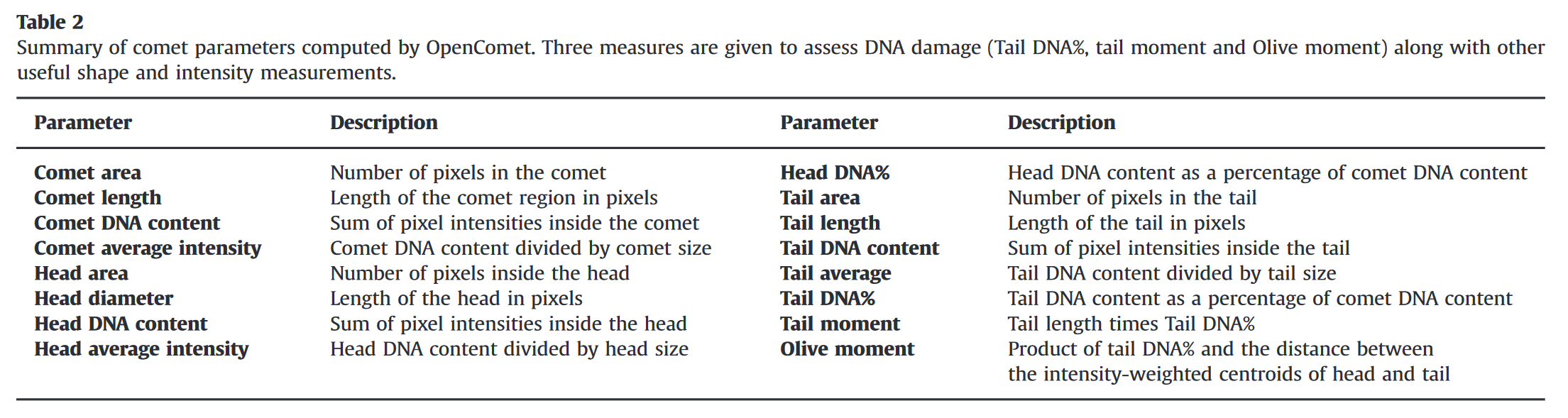


Gyori BM, Venkatachalam G, Thiagarajan PS, Hsu D, Clement M-V. 2014. OpenComet: an automated tool for comet assay image analysis. Redox biology 2:457–65.
